# Understanding Curriculum Implementation with Entrustable Professional Activities Through the Lens of Normalization Process Theory

**DOI:** 10.5334/pme.2440

**Published:** 2026-06-11

**Authors:** Marije P. Hennus, Roberta I. Ladenheim, H. Carrie Chen, Fremen Chihchen Chou, Stanley J. Hamstra, Eva K. Hennel, Adrian P. Marty, Gustavo S. Romao, Mabel Yap, Olle ten Cate

**Affiliations:** 1 Department of Pediatric Intensive Care, University Medical Center Utrecht, Utrecht, The Netherlands; 2 Universidad del Hospital Italiano de Buenos Aires, Argentina; 3 Department of Health Systems Science, Kaiser Permanente Bernard J. Tyson School of Medicine, Pasadena, California, United States; 4 China Medical University, Taiwan; 5 School of Medicine and Center for Faculty Development, China Medical University Hospital, Taichung, Taiwan; 6 Department of Surgery, University of Toronto, Toronto, Canada; 7 Holland Bone and Joint Program, Sunnybrook Research Institute, Toronto, Canada; 8 Swiss Institute for Medical Education, Bern, Switzerland; 9 Department of Anesthesiology, Intensive Care Medicine and Pain Therapy, University Hospital Balgrist, Zurich, Switzerland; 10 University of Ribeirão Preto/Febrasgo, Ribeirão Preto, São Paulo, Brazil; 11 Professional Training and Assessment Standards, Ministry of Health, Singapore; 12 Education Office, National University Singapore Health System, Singapore; 13 Medical Education at University Medical Center Utrecht, The Netherlands

## Abstract

Competency-based medical education (CBME) is widely adopted in numerous countries, but many programs struggle to translate its conceptual promises into routine educational and clinical practice. Entrustable Professional Activities (EPAs), a key mechanism to operationalize CBME, have been implemented in different ways with substantial variation across contexts. While implementation challenges are well described, the field lacks explanatory approaches to understand why such variation occurs and how it shapes practice.

In this *Show-and-Tell* contribution we apply Normalization Process Theory (NPT), an implementation theory, to analyze how EPA-based curriculum reforms are taken up, enacted, and sustained across settings.

We conducted a comparative, theory-informed analysis of implementation experiences from five regions (in Latin America, Asia, North America, Europe) drawing on materials from an international EPA symposium (Barcelona, 2025). Data sources include a pre-symposium survey, invited presentations, transcripts of plenary discussions, and follow-up reflections. Using NPT’s four core constructs (coherence, cognitive participation, collective action, and reflexive monitoring), we examined how stakeholders make sense of EPAs, engage with implementation, integrate practices into clinical routines, and adapt them over time.

The analysis revealed recurring mechanisms shaping implementation across contexts. Challenges included conceptual ambiguity, variable engagement, workflow pressures, and misalignment with existing practices. At the same time, enabling conditions emerged across settings: clear communication of purpose, phased implementation strategies, coordinated faculty development, leadership support, and context-sensitive adaptation. By foregrounding implementation mechanisms rather than outcomes alone, NPT offers a transferable analytic lens to guide, evaluate, and refine curriculum reforms.

## Background and Need to Understand Implementation Practices

Competency-based medical education (CBME) has become an influential reform agenda in health professions education (HPE), aiming to shift programs from time-based to outcome-oriented learning and assessment [1]. Several countries and institutions have chosen to implement Entrustable Professional Activities (EPAs) as a CBME component but many programs also struggle with the practical translation of CBME and EPA principles into routine clinical teaching and supervision [2]. These challenges arise not only at the clinical interface but also at the curricular, institutional, and regulatory levels, where existing structures, established routines, and professional norms can limit the ability to enact meaningful change [3]. As a result, EPA-based reforms may only be partially realized. Educators continue to seek ways to bridge the gap between conceptual models and day-to-day implementation.

EPAs were introduced to operationalize CBME by offering task-based units of professional work that connect abstract competency frameworks to the realities of clinical practice [4,5]. Over the past decade, EPA-based approaches have been implemented globally [6], nationally [7,8,9] and locally across undergraduate and postgraduate programs across various health professions [10,11]. While detailed guidelines for EPA development and implementation exist [12,13,14,15], their implementation appears to vary considerably across systems and institutions. In particular, the core feature of EPAs, i.e. to grant increasing autonomy in patient care through entrustment decisions, is not always well understood or operationalized [16]. Relatedly, the distinction between individual ad hoc entrustment decisions in daily practice and summative entrustment decisions in teams such as clinical competency committees (CCCs) is not always operationalized [17]. Programs implementing EPAs report conceptual ambiguity, inconsistent faculty engagement, workload constraints, questions about assessment fidelity, and difficulty embedding EPA practices within clinical workflows [2,18]. Together, these recurring challenges suggest that difficulties in EPA implementation stem not merely from framework design, but from the work required by educational, clinical, and organizational actors to make sense of EPAs, integrate them into practice, and sustain them over time. While variability in EPA implementation is widely reported, the field lacks explanatory frameworks that can account for why such variation occurs and how it shapes implementation processes. This gap limits the ability to anticipate challenges and design context-sensitive implementation strategies, underscoring the need for theory-informed approaches.

Current approaches to studying curriculum implementation often rely on descriptive accounts that are closely tied to specific contexts, limiting their ability to generate transferable insights across settings. Although there is increasing recognition of the value of implementation science [19,20,21], there remains a lack of structured approaches that can systematically examine how educational practices are enacted, sustained, and embedded in routine work across diverse environments. Normalization Process Theory (NPT) [22,23,24], with its focus on the processes through which new practices become normalized, offers a promising lens for interrogating these dynamics. However, its use in medical education has not been consistently translated into a practical analytic approach that supports comparative understanding across contexts. This creates an opportunity to explore how such a theory might be operationalized to move beyond context-bound description toward more explanatory and transferable insights.

## Goal of the Innovation

This work seeks to operationalize NPT as a practical analytic approach for examining curriculum implementation across diverse international contexts. The innovation lies in applying the theory to demonstrate how its constructs can be systematically translated into analytic strategies that enable comparison across cases and illuminate patterns that are otherwise difficult to discern. Through this approach, we aim to show how theory can be used as a tool for generating deeper, more structured insights into implementation processes, while offering a model that others may adapt in studying complex educational practices.

### Normalization Process Theory

NPT is an implementation theory developed by May [23,25], offering a framework for understanding how complex interventions become embedded (“normalized”) in everyday practice. Rather than focusing on whether an intervention is effective, NPT attends to the work required by individuals and groups to implement, sustain, and adapt new practices within existing social and organizational systems. By foregrounding this implementation work, NPT enables an analysis of how stakeholders make sense of, engage with, enact, and appraise innovations such as EPA-based curricula within clinical learning environments.

**Figure 1:**
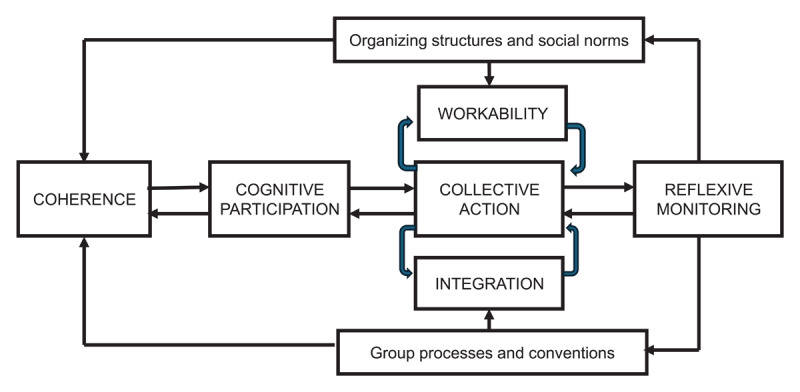
The *Implementation Core* of Normalization Process Theory (from May et al 2020).

NPT identifies four core constructs that shape the work required for implementation: Coherence (the extent to which a practice is well understood and made meaningful), Cognitive Participation (the relational work of building and sustaining engagement of individuals and groups), Collective Action (the operational work of enacting a practice in context), and Reflexive Monitoring (the appraisal and adaptation of a practice over time). Each construct includes subconstructs that offer further analytic granularity. These constructs provide an analytic lens to examine how implementation unfolds in practice and to identify mechanisms underlying variation across contexts. The core constructs are summarized in Figure 1 and further elaborated, based on prior literature [24,25,26,27], in Table 1.

**Table 1:** Core Constructs and Contextual Features of Normalization Process Theory.


CONTEXT FEATURES	

Transactional space (the social, organizational, and institutional setting where implementation work unfolds), including the capacity for adaptive execution, strategic coordination, and negotiation. Social norms and roles (shared expectations, professional identities, and informal rules that shape what is considered appropriate or legitimate). Social cognitive resources (the collective intentionality, commitment, and preparedness of individuals and teams to engage with change). Material and informational resources (availability of funding, technologies, artifacts, documentation, and communication channels to support implementation).	

**CORE CONSTRUCTS**	**EXPLANATION AND SUBCONSTRUCTS**

**Coherence** (the apparent qualities of a proposed new practice: does it make sense?)	Coherence refers to the extent to which a new practice can be understood and made meaningful by individuals and groups. It reflects how stakeholders differentiate it from existing practices, understand its purpose, and internalize its value. Subconstructs are: **Differentiation** (*I can see how it differs from usual ways of working*) **Communal specification** (*Staff in this organization have a shared understanding of the purpose*) **Individual specification** (*I understand how it affects the nature of my own work*) **Internalization** (*I can see the potential value for my work*)

**Cognitive Participation** (the engagement of individuals and groups)	Cognitive Participation refers to the relational work of building and sustaining commitment to a new practice. It is a property of individuals and groups, and includes activating leadership, enrolling others, legitimizing roles, and sustaining engagement. Subconstructs are: **Initiation** (*Key people drive the change forward and get others involved*) **Enrolment** (*I’m open to working with colleagues in this new way*) **Legitimation** (*I believe that participating is a legitimate part of my role*) **Activation** (*I will continue to support this*)

**Collective Action** (the actual agency in context)	Collective Action describes the concerted effort to integrate a new practice into existing workflows and is a property of the community and the system. It reflects whether people are capable, supported, and organized to implement the change. Subconstructs are: **Skill-set workability** (*The work is assigned to individuals with appropriate skills sets; sufficient training is provided*) **Interactional workability** (*I can easily integrate this into my work*). **Relational Integration** (*This may disrupt existing working relationships, but I have confidence in other people’s ability to adapt*) **Contextual integration** (*Sufficient resources and management support are available*)

**Reflexive Monitoring** (appraisal of a new practice and recon-figuration if needed)	Reflexive Monitoring refers to how people assess and reflect on the effects of the new practice and adjust over time. It includes both individual and collective forms of evaluation. Subconstructs are: **Systematization** (*I am aware of reports about the effects of the intervention*) **Communal appraisal** (*The staff agree that this is worthwhile*) **Individual appraisal** (*I value the effects it has had on my work*) **Reconfiguration** (*Feedback is used for future improvement; I can modify how I work with it*)


Importantly, the four core constructs are shaped by the readiness of the context in which the change must happen. May et al. explain how contextual readiness includes social norms, social cognitive resources, and material and informational resources, in addition to a transactional space [25]. These contextual features influence whether, how, and to what extent NPT mechanisms can be activated in practice. This view is further expanded by Hillis et al., who describe workability and integration as key contextual conditions that influence whether and how practices become normalized [26]. In our study, contextual factors are considered in relation to the way they enable or constrain the operation of NPT mechanisms across cases. The statements in italics in Table 1 are derived from NPT’s *NoMad* measurement tool [27] and are included to support the interpretation of the constructs.

## Steps Taken for Innovation: Application of NPT

Rather than producing generalizable claims about individual systems, the analysis provides a comparative synthesis of implementation mechanisms. By systematically examining how EPA practices are made meaningful, enacted, and adapted across systems, this approach offers transferable theory-informed insights that can support educators in guiding, evaluating, and refining EPA-based curriculum reforms in diverse settings. This work also positions NPT not only as an organizing framework, but as an analytic lens that enables identification of mechanisms underlying variation in implementation across settings.

### International case studies as source materials

To develop a theory-informed understanding of EPA implementation across contexts, we undertook a multi-source analytic approach grounded in NPT. The work drew on materials generated before, during, and after a 2025 Barcelona invitational symposium, which brought together 59 participants with experience in EPA-based curricular reform across diverse contexts from 19 countries (see supplemental data). Rather than presenting individual case histories (available in the supplemental materials), our aim was to identify recurring implementation mechanisms across contexts, not to produce exhaustive or representative accounts of individual systems. This approach constitutes a theory-informed, interpretive synthesis rather than a formal qualitative study. A more extensive report of implementation experiences is available elsewhere [28].

### Data sources

Data sources included a pre-symposium survey, five invited regional or national case presentations, plenary discussion transcripts, and post-symposium reflections by the core author team. The pre-symposium survey invited participants to reflect on challenges and lessons learned in EPA implementation. Responses were summarized and synthesized into an overview to inform symposium discussions. Five cases (Singapore, Switzerland, Taiwan, North America (Canada and USA) and Latin America (with a focus on Argentina and Brazil) were purposefully selected to maximize variation in health system organization, regulatory environments, cultural norms, and stages of EPA adoption. Presentations provided structured insights into national priorities and challenges and stimulated a facilitated discussion. Post-meeting reflections by the core author team (MPH, OtC, RIL) added contextual interpretation but were treated cautiously due to their retrospective and interpretive nature. Case descriptions were subsequently developed (OtC) and validated with presenters. This sampling strategy enabled comparison across diverse contexts while maintaining sufficient coherence for cross-case analysis.

### Development of a shared analytic framework

We developed a shared analytic framework, centered on the four core NPT constructs and on contextual preconditions identified in NPT literature including social norms, cognitive resources, material and informational infrastructures, and the transactional space in which implementation work unfolds. This framework was used as a sensitizing guide, supported by a structured template to ensure consistent attention to each construct across cases.

### Iterative application of NPT constructs to each case

Each case was reviewed by the core author team using the NPT framework, examining how: (a) participants made sense of EPA implementation within their context *(coherence)*; (b) individuals and groups mobilized to support the work *(cognitive participation*); (c) EPA practices were enacted within clinical and institutional routines *(collective action);* and (d) stakeholders evaluated and adapted implementation efforts over time (*reflexive monitoring)*. Iterative discussions were used to refine interpretations, align applications of the constructs, and surface potential interpretive biases associated with authors’ own institutional or national contexts.

### Construction of a cross-case synthesis

Emergent insights were integrated into a comparative synthesis structured around NPT’s core constructs (Table 2), linking each construct to recurring cross-context patterns and illustrative examples. The synthesis prioritizes mechanisms observed across multiple sources while acknowledging variation in depth of evidence across cases. Through this structured, theory-informed, and collaborative analytic approach, we transformed these insights into a coherent model for understanding EPA implementation across diverse settings. The resulting synthesis is therefore intended not as a definitive account of each national or regional system, nor a comprehensive current snapshot, but rather as an analytic demonstration of how NPT can be applied to identify implementation mechanisms across varied contexts.

**Table 2:** Cross-context synthesis of EPA implementation using NPT.


NPT CORE CONSTRUCT	CROSS-CONTEXT PATTERNS AND EXAMPLES

**Coherence** (the apparent qualities of a proposed new practice: does it make sense?)	**Latin America**: EPAs provided an accessible entry point where “competence” was ideologically resisted, improving initial coherence despite lack of a CBME foundation, supporting early uptake but with limited conceptual depth. **Singapore**: Strong coherence via a shared national vision and mental model, supported by dual top-down and bottom-up strategies, facilitating aligned implementation and sustained engagement. **Taiwan**: EPA adoption was advanced through specialty-led initiatives and visible tools (e.g., EMYWAY). However, alignment regarding the formative and summative purposes of EPA use remained variable across settings, contributing to ongoing conceptual and operational challenges. **Canada**: Reflexive monitoring revealed limited coherence in practice despite initial conceptual clarity, prompting recalibration, highlighting the need for ongoing alignment to support implementation fidelity. **Switzerland**: Weak coherence; EPAs often misperceived as mere assessment tools, leading to conceptual confusion and resistance undermining engagement and slowing implementation.

**Cognitive Participation** (the engagement of individuals and groups)	**Latin America**: CP supported through regional EPA courses and community building, despite lack of formal mandates, enabling initial engagement but with variable sustainability. **Singapore**: Strong CP via national faculty development, EPA champions, and high-level stakeholder coordination, supporting broad engagement and role legitimation. **Taiwan**: Partial CP; faculty and residents engaged locally, but national coordination and clear role legitimation were insufficient, limiting broader sustained collective commitment. **USA**: CP occurred at specialty and institutional levels; absence of national mandate limited broad engagement, resulting in fragmented implementation efforts. **Switzerland**: CP hampered by weak value proposition; clinicians questioned relevance and legitimacy of the reform, limiting buy-in and engagement.

**Collective Action** (the actual agency in context)	**Latin America**: CA largely informal and partial; absence of integrated tech or shared infrastructure, limiting workflow integration and alignment. **Singapore**: Strong CA via coordinated leadership, tech platforms, and alignment with service needs (e.g., stackable EPAs), facilitating integration into routine practice. **Taiwan**: CA was facilitated by digital tools such as EMYWAY. Nevertheless, variation in CCC processes, supervisory practices, and role clarity limited interactional workability and consistency across implementation settings. **Canada**: CA challenged by residents’ responsibility to initiate assessments and program variability; potential misalignment of assessment burden, leading to misalignment with workflow and assessment burden concerns. **Switzerland**: Some tools available, but weak engagement and misalignment with local workflows limited enactment, constraining effective implementation.

**Reflexive Monitoring** (appraisal of a new practice and reconfiguration if needed)	**Latin America**: Incremental reforms and faculty reflection led to tangible shifts (e.g., longer rotations, more feedback), despite lack of systematization, supporting gradual adaptation but limiting scalability. **Singapore**: Ongoing refinement, informed by feedback from pilots and service needs; RM was explicitly embedded, supporting continuous improvement and sustainability. **Taiwan**: Early digital dashboards enabled feedback loops (e.g., EMYWAY), though summative vs formative tensions persisted, shaping adaptation but maintaining conceptual challenges. **Canada**: National monitoring by RCPSC triggered course correction, including specialty-specific adaptations, supporting system-level recalibration. **Switzerland**: Some macro-level RM (e.g., questioning whether EPAs alone can shift educational culture), but limited evidence of structured evaluation systems, constraining feedback loops and adaptation.


## Outcomes of Innovation: What Did the NPT Lens Enable?

Analysis of the five EPA implementation cases through an NPT lens revealed shared mechanisms as well as context-specific variations in how EPA-based curricula were taken up, enacted, and sustained. Examining the cases through NPT’s four constructs enabled a more coherent and theory-informed interpretation of implementation patterns than was possible from descriptive accounts alone. Table 2 presents a cross-case synthesis structured by NPT constructs, illustrating how implementation patterns manifest across contexts. It highlights how meaning-making, engagement, enactment, and appraisal processes vary across settings and how contextual conditions shape these mechanisms. These patterns are likely recognizable to educators working in other settings where conceptual ambiguity, variable engagement, and workflow constraints similarly shape implementation efforts.

### Coherence: meaning making unfolded

Across cases, stakeholders varied in how they understood the purpose and value of EPAs. Singapore demonstrated strong coherence, supported by a shared national vision, transparent communication, coordinated faculty development and resources aligned with the intended role of EPAs. In Switzerland and parts of North America, coherence was weaker, as EPAs were sometimes understood primarily as assessment checklists without consequences for entrustment, rather than as units of professional practice for gradual increase of autonomy, which caused conceptual ambiguity. Coherence gaps shaped subsequent implementation challenges: when supervisors and trainees lacked a shared understanding of what EPAs were intended to accomplish, this uncertainty influenced how they viewed supervision, learning trajectories, and the relevance of EPAs in daily clinical work, resulting in feelings of unnecessary burden. Canada’s rapid embrace of EPAs in their Competence-By-Design model is currently being reconsidered [9].

### Cognitive participation: the relational work of engagement

Patterns of engagement differed markedly across cases. Singapore and Taiwan benefited from early leadership, structured faculty development, and local champions, which helped legitimize EPA-related work. In contrast, Latin America, Switzerland, and parts of the United States showed more variable engagement, often relying on volunteer faculty or small expert groups. The level of engagement and how this was legitimized were highly sensitive to workload pressures and institutional support. In several cases, stakeholders hesitated to commit because the benefits of EPAs were not clearly articulated or because participation did not align with perceived professional roles. These conditions left cognitive participation fragile and easily disrupted by leadership turnover or shifting priorities.

### Collective action: enactment in real settings and workflow

Substantial variation was observed in how EPA practices were integrated into everyday clinical routines. Singapore and Taiwan showed the strongest collective action, supported by coordinated leadership structures, integrated digital platforms, and clear operational procedures. For example, one discipline in Taiwan developed a digital platform, which was quickly tested by some others and subsequently endorsed by the accreditation agency and finally used widely across Taiwan to improve the workflow for clinical educators. What still remained however, were gaps in CCC implementation and supervision structures. In contrast, Canada showed friction in everyday enactment, partly due to residents initiating most assessments and program-level variability. Switzerland and Latin America faced infrastructure limitations, inconsistent access to digital systems, and variable supervisory practices, which hindered alignment with clinical workflows. Across cases, collective action was most effective when earlier coherence and cognitive participation work had already created a clear rationale, shared language, and relational support for new practices. Across contexts, lack of time and resources constrained enactment, with similar challenges reported across both low-resource and high-resource settings.

### Reflexive monitoring: appraisal and adaptation over time

Mechanisms for evaluating and adjusting EPA implementation varied widely. Taiwan used early digital dashboards to make patterns of assessment and feedback visible, while Singapore embedded structured review processes into its national rollout. In Latin American countries and Switzerland, evaluation was largely informal, occurring through local faculty discussions rather than systematic monitoring. Canada and the United States demonstrated mixed approaches, combining national review processes with local adaptations, though variability across institutions limited coherence. Where coherence and collective action were stronger, reflexive monitoring was more likely to produce meaningful feedback loops supporting adaptation; where earlier mechanisms were weak, evaluation tended to be sporadic and less actionable. These findings suggest that reflexive monitoring is shaped by the strength of preceding implementation processes.

### Interaction of NPT mechanisms across contexts

Although the NPT constructs are analytically distinct, the cases demonstrated their dynamic interdependence. Limited coherence, such as unclear differentiation between EPAs and traditional assessment tools, often undermined cognitive participation by making it difficult for educators to see the value of engaging with EPA practices. Fragile engagement, in turn, constrained collective action, as insufficient buy-in or role clarity reduced the likelihood that EPA processes would be integrated into clinical routines. Weak collective action diminished opportunities for reflexive monitoring, since inconsistent enactment produced limited or fragmented data to support meaningful appraisal. Conversely, settings in which coherence was deliberately established early and widely communicated to build engagement were better able to develop feasible workflows and iterative evaluation structures. Together, these patterns suggest that EPA implementation depends not on isolated activities but on the alignment and reinforcement of mechanisms across all four NPT constructs.

## Critical Reflection

The use of NPT as the overarching analytic frame brought several strengths to this comparative synthesis. First, the theory provided a coherent set of sensitizing constructs that helped to explain how EPA implementation unfolded across diverse contexts [23,24]. By applying the same analytic lens to heterogeneous data, the analysis moved beyond surface-level descriptions and made visible the mechanisms shaping adoption, enactment, and sustainability. NPT also highlighted phenomena often implicit in implementation narratives, such as how coherence gaps can cascade into relational fragility or workflow misalignment. Third, by emphasizing mechanisms rather than outcomes, the framework demonstrated its transferability across settings.

At the same time, the analysis exposed challenges related to combining and interpreting heterogeneous data across countries and contexts. Because each case was analysed against different types of material, including symposium presentations, pre-meeting surveys, and follow-up reflections, the variable richness and granularity of the data influenced the level of insight that was possible for particular mechanisms. Some NPT subconstructs, such as relational integration within collective action or systematization within reflexive monitoring, appeared less visible not because they lacked importance, but because available materials offered limited insight into the day-to-day work of implementing EPA processes. This reflects a broader challenge in implementation research, where incomplete documentation can obscure influential mechanisms or make it difficult to discern whether absences reflect practice or reporting gaps [20,21]. In response, the analysis prioritized cross-case signals supported by multiple data sources and avoided overinterpretation of sparsely evidenced patterns. Another tension concerned interpretive negotiation within a diverse author team representing different countries, health systems, and stages of EPA maturity. While this diversity enriched the analysis, it also required deliberate reflexivity to distinguish between empirical signals and interpretations shaped by authors’ own contexts. A further limitation relates to NPT’s analytic scope. Although the theory offers a powerful lens for examining how innovations become embedded in routine practice, it attends more closely to micro- and meso-level enactment than to macro-level forces such as accreditation policies, national workforce structures, or sociopolitical histories of medical training. Several cases highlighted these broader influences, which sit partly outside NPT’s explanatory range. To account for this, we incorporated contextual features alongside NPT constructs in the synthesis table, enabling a more complete understanding without overstretching the theory.

Despite these limitations, applying NPT enhanced both the rigor of the synthesis and the implications for practical utility. The framework helped identify cross-setting configurations of context, mechanism, and outcome that would otherwise remain obscured, supporting the development of insights that are both theoretically grounded and actionable. NPT also underscored the importance of examining how stakeholders negotiate, enact, and adapt EPA practices within complex clinical learning systems.

Across cases, these findings indicate that implementation unfolds through the interaction of multiple mechanisms. Coherence shaped subsequent engagement and enactment processes: where stakeholders developed a shared understanding of purpose and value, implementation trajectories were more stable, whereas weak coherence was associated with conceptual drift and fragile downstream processes. Cognitive participation emerged as dependent on both conceptual clarity and alignment with professional roles and organizational conditions, with engagement strengthened by leadership, faculty development, and local champions, and constrained by competing clinical demands and unclear expectations. Collective action was shaped by the interaction between design features and contextual conditions, as feasible workflows, digital tools, and supervisory structures supported integration, while misalignment with clinical routines limited enactment. Reflexive monitoring influenced sustainability, with structured feedback processes enabling adaptation and course correction, and fragmented evaluation limiting learning from implementation. Across all cases, contextual conditions, including regulatory environments, professional cultures, and resource availability, shaped the extent to which these mechanisms could be activated.

Taken together, the findings indicate that EPA implementation unfolds through the alignment and reinforcement of mechanisms across all four NPT constructs, rather than through isolated interventions. Applying Normalization Process Theory across diverse EPA implementation contexts demonstrates how meaning-making, engagement, workflow integration, and appraisal interact to shape implementation trajectories. This mechanism-focused lens moves beyond descriptive case accounts and enables a theory-informed understanding of variation across contexts. By demonstrating how NPT supports both interpretation and informed implementation, this analysis provides a transferable approach that educators and leaders can use to design, evaluate, and refine EPA implementation in their own settings. This work provides a starting point for more systematic, theory-informed implementation research in health professions education, particularly studies that prospectively examine how implementation mechanisms evolve across contexts and over time.

## Additional File

The additional file for this article can be found as follows:

10.5334/pme.2440.s1 Supplemental material 1. 5 Case studies.

## References

[1] Frank JR, Snell LS, ten Cate O, Holmboe ES, Carraccio C, Swing SR, et al. Competency-based medical education: theory to practice. Med Teach. 2010;32(8):638–645. DOI: 10.3109/0142159X.2010.501190 20662574

[2] Caverzagie KJ, Nousiainen MT, Ferguson PC, ten Cate O, Ross S, Harris KA, et al. Overarching challenges to the implementation of competency-based medical education. Med Teach. 2017;39(6):588–593. DOI: 10.1080/0142159X.2017.1315075 28598747

[3] Van Melle E, Frank JR, Holmboe ES, Dagnone D, Stockley D, Sherbino J, et al. A core components framework for evaluating implementation of competency-based medical education programs. Acad Med. 2019;94(7):1002–1009. DOI: 10.1097/ACM.0000000000002743 30973365

[4] ten Cate O. Entrustability of professional activities and competency-based training. Med Educ. 2005;39(12):1176–1177. DOI: 10.1111/j.1365-2929.2005.02341.x 16313574

[5] ten Cate O. Trust, competence, and the supervisor’s role in postgraduate training. BMJ. 2006;333(7571):748–751. DOI: 10.1136/bmj.38938.407569.94 17023469 PMC1592396

[6] Fitzpatrick S, editor. Global competency and outcomes framework for universal health coverage. Geneva: World Health Organization; 2022.

[7] de Graaf J, Bolk M, Dijkstra A, van der Horst M, Hoff RG, ten Cate O. The implementation of entrustable professional activities in postgraduate medical education in The Netherlands: rationale, process, and current status. Acad Med. 2021;96(7 Suppl):S29–S35. DOI: 10.1097/ACM.0000000000004110 34183599

[8] Hennus MP, Nusmeier A, van Heesch GGM, Riedijk MA, Schoenmaker NJ, Soeteman M, et al. Development of entrustable professional activities for paediatric intensive care fellows: a national modified Delphi study. PLoS One. 2021;16(3):e0248565. DOI: 10.1371/journal.pone.0248565 33735195 PMC7971696

[9] Cheung WJ, Bhanji F, Gofton W, Hall AK, Karpinski J, Richardson D, et al. Design and implementation of a national program of assessment model integrating entrustable professional activity assessments in Canadian specialist postgraduate medical education. Perspect Med Educ. 2024;13(1):44–55. DOI: 10.5334/pme.956 38343554 PMC10854461

[10] Pinilla S, Lenouvel E, Cantisani A, Klöppel S, Strik W, Huwendiek S, et al. Working with entrustable professional activities in clinical education in undergraduate medical education: a scoping review. BMC Med Educ. 2021;21(1):172. DOI: 10.1186/s12909-021-02608-9 33740970 PMC7980680

[11] O’Dowd E, Lydon S, O’Connor P, Madden C, Byrne D. A systematic review of 7 years of research on entrustable professional activities in graduate medical education, 2011–2018. Med Educ. 2019;53(3):234–249. DOI: 10.1111/medu.13792 30609093

[12] ten Cate O, Chen HC, Hoff RG, Peters H, Bok H, van der Schaaf M. Curriculum development for the workplace using entrustable professional activities (EPAs): AMEE guide No. 99. Med Teach. 2015;37(11):983–1002. DOI: 10.3109/0142159X.2015.1060308 26172347

[13] Peters H, Holzhausen Y, Boscardin C, ten Cate O, Chen HC. Twelve tips for the implementation of EPAs for assessment and entrustment decisions. Med Teach. 2017;39(8):802–807. DOI: 10.1080/0142159X.2017.1331031 28549405

[14] Hennus MP, Jarrett JB, Taylor DR, ten Cate O. Twelve tips to develop entrustable professional activities. Med Teach. 2023;45(7):701–707. DOI: 10.1080/0142159X.2023.2197137 37027517

[15] ten Cate O, Taylor DR. The recommended description of an entrustable professional activity: AMEE guide No. 140. Med Teach. 2021;43(10):1106–1114. DOI: 10.1080/0142159X.2020.1838465 33167763

[16] ten Cate O, Schumacher DJ. Entrustable professional activities versus competencies and skills: exploring why different concepts are often conflated. Adv Health Sci Educ Theory Pract. 2022;27(2):491–499. DOI: 10.1007/s10459-022-10098-7 35226240 PMC9117349

[17] ten Cate O, Burch VC, Chen HC, Chou FC, Hennus MP, editors. Entrustable professional activities and entrustment decision-making in health professions education. London: Ubiquity Press; 2024. DOI: 10.5334/bdc 41401262

[18] Sohrmann M, Berendonk C, Nendaz M, Bonvin R; Swiss Working Group for Profiles Implementation. Nationwide introduction of a new competency framework for undergraduate medical curricula: a collaborative approach. Swiss Med Wkly. 2020;150:w20201. DOI: 10.57187/smw.2020.20201 32294223

[19] Gale R, Grant J. AMEE medical education guide No. 10: managing change in a medical context: guidelines for action. Med Teach. 1997;19(4):239–249. DOI: 10.3109/01421599709034200

[20] Nilsen P. Making sense of implementation theories, models and frameworks. Implement Sci. 2015;10:53. DOI: 10.1186/s13012-015-0242-0 25895742 PMC4406164

[21] Damschroder LJ, Aron DC, Keith RE, Kirsh SR, Alexander JA, Lowery JC. Fostering implementation of health services research findings into practice: a consolidated framework for advancing implementation science. Implement Sci. 2009;4:50. DOI: 10.1186/1748-5908-4-50 19664226 PMC2736161

[22] May C, Finch T. Implementing, embedding, and integrating practices: an outline of normalization process theory. Sociology. 2009;43(3):535–554. DOI: 10.1177/0038038505052497

[23] May C. Towards a general theory of implementation. Implement Sci. 2013;8:18. DOI: 10.1186/1748-5908-8-18 23406398 PMC3602092

[24] Murray E, Treweek S, Pope C, MacFarlane A, Ballini L, Dowrick C, et al. Normalisation process theory: a framework for developing, evaluating and implementing complex interventions. BMC Med. 2010;8:63. DOI: 10.1186/1741-7015-8-63 20961442 PMC2978112

[25] May C, Finch T, Rapley T. Normalization process theory. In: Nilsen P, Birkin S, editors. Handbook on implementation science. Cheltenham, UK: Edward Elgar Publishing; 2020. DOI: 10.4337/9781788975995.00013

[26] Hillis A. Applying normalization process theory. In: Nilsen P, editor. Implementation science: theory and application. London: Routledge; 2024. pp. 223–236. DOI: 10.4324/9781003318125-21

[27] Finch TL, Girling M, May CR, Mair FS, Murray E, Treweek S, et al. NoMAD: implementation measure based on normalization process theory [Internet]. Newcastle upon Tyne: Newcastle University; 2015 [cited 2025 Oct 23]. Available from: https://dissemination-implementation.org/wp-content/uploads/2024/08/NoMAD_Instrument.pdf.

[28] ten Cate O, Ladenheim RI, Chen HC, Chou FC, Hamstra SJ, Hennel EK, et al. Implementation of curricula with entrustable professional activities: issues, challenges and lessons learned, from an international perspective. In: Kwan B, Nolan J, Schultz K, McEwen L, Dalgarno N, editors. Competency-based medical education. Amsterdam: Elsevier; 2026. Forthcoming.

