# Supplementary material for: Understanding Curriculum Implementation with Entrustable Professional Activities Through the Lens of Normalization Process Theory
Source: Perspect Med Educ. 2026 Jun 11;15(1):502–11. doi: 10.5334/pme.2440 (PMC13262643; doi:10.5334/pme.2440)
Supplement: Supplemental material 1. — 5 Case studies. [file pme-15-1-2440-s1.pdf]

Supplemental materials: five case studies

Five presentations were provided from Latin America, Asia (Singapore and Taiwan), North America (Canada and USA combined) and Europe (Switzerland). While continental differences are impossible to report because of the large differences between countries within each continent, the examples chosen, show a breadth of intercontinental experiences. We reviewed these with reference to the Normalization Process Theory module. These descriptions must be viewed as eclectic snapshots, presented during the symposium. They are no more and no less than examples that illustrate the implementation issues, challenges and lessons learned, summarized above.

1. **Latin America.** EPA implementation in Latin America has been largely unsystematic, spanning both undergraduate and postgraduate levels. It has been driven by diverse stakeholders—universities, professional associations, hospitals, and governmental bodies—often without coordination or alignment with broader competency-based reforms. In many cases, EPA sets were imported or translated with minimal contextual adaptation, sometimes of limited quality, which compromised their fit with local practice. Despite these challenges, EPAs have provided a pragmatic entry point to CBME, particularly in contexts where the concept of "competence" faced ideological resistance.

   In a region where EPAs were introduced without a strong CBME foundation, their implementation revealed that some key elements were necessary to support genuine change: continuity of rotations with sustained tutor supervision, a clearer recognition of the teaching role and the value of workplace-based learning, and the importance of consistent, longitudinal assessment practices. The effort to implement EPAs helped bring these aspects to the forefront, leading to changes that are laying a stronger foundation for future EPA-based reforms. Faculty-development initiatives—especially the regional online EPA courses in Spanish and Portuguese—have played a central enabling role, helping to build shared understanding, foster regional networks, and gradually shift educational culture among supervisors and trainees. Even before full curricular reform, early adoption brought tangible benefits: greater attention to workplace observation and feedback, more explicit discussion of trainee progression, modest reforms such as longer clinical rotations and strengthened supervision, and the introduction of logbooks and digital tools to support assessment practices.

Viewed through the NPT framework, the Latin American experience reflects how constraints within the *Context* —including limited integration of health and education

sectors, weak accreditation mechanisms, and long-standing time-based, teacher-centered traditions—have challenged the *Interactional Workability and Integration (Collective Action)* necessary for sustained change. EPAs nevertheless offered a more concrete and less ideologically contested focus for *Coherence*, enabling stakeholders to recognize the gap between graduates' preparedness for practice and existing educational models. *Cognitive Participation* was fostered through regional faculty-development initiatives and by framing EPAs as a way to structure and legitimize supervisors' existing informal entrustment practices. However, *Collective Action* has often been partial: assessment remains fragmented, entrustment decisions are rarely formalized, and programmatic alignment among outcomes, teaching, and evaluation is still limited. Early efforts at *Reflexive Monitoring*—through logbooks, digital tools, and faculty discussions—have begun to reshape supervision practices and stimulate curriculum reform, yet systematic evaluation and continuous adaptation remain incipient. Overall, the region illustrates how even small, theory-guided initial steps can catalyze broader conversations and incremental reforms, while also highlighting the risk of superficial implementation when change is not supported by coherent frameworks, faculty preparation, and sustained monitoring.

2. **Singapore.** Singapore's healthcare system faces significant challenges from an ageing population and rising prevalence of chronic diseases, while being confronted by a shrinking workforce pipeline, and implementation of new care models. To address these evolving healthcare needs, Singapore has embarked on a journey to implement competency-based education, anchored by EPAs, aiming to train the right number of healthcare professionals with the right competencies needed to support national health care strategies.
   The EPA implementation at a national level consists of three broad phases – (i) initial capability-building from 2017 to 2019, (ii) implementing EPA pilot for selected disciplines in medical, nursing, and pharmacy from 2019 to 2020, and (iii) ongoing efforts to scale the use of EPAs across the continuum of education and beyond formal education. Throughout all three phases, change management and faculty development remain cornerstone elements.
   As of October 2025, more than 490 national-level EPAs have been developed across 65 training programmes spanning various healthcare disciplines and professions, with over 4,500 healthcare trainees trained and assessed using EPAs. To sustain successful large-scale EPA implementation, Singapore employed a dual strategy: (i) top-down approaches, where the Ministry of Health integrated EPAs into

programmes' training standards, collaborated with professional boards to incorporate EPAs into graduation and registration requirements, and continuously enhanced the national Faculty Development Framework, and (ii) ground-up approaches through EPA champions appointed within training institutions to spearhead EPA initiatives, deliver in-house workshops, curate training resources, and develop faculty members and administrators.

Beyond formal training, EPAs are used for continuing education and professional development in Singapore. Healthcare professionals complete modular, stackable, on-the-job training anchored on EPAs to take on roles not traditionally performed by them (e.g., collaborative prescribing by advanced practice nurses and pharmacists). Through 'stackable EPAs', healthcare professionals can be trained for new roles based on service needs in an agile manner, thereby increasing the fluidity of roles within and across professions. The envisioned outcome is healthcare teams with complementary skills across professions, with individuals possessing a dynamic portfolio of EPAs for which they are qualified.

The implementation of EPAs in Singapore was and is not possible without the support from the international EPA community. The international online course 'Ins and Outs of EPAs' has been highly instrumental and was attended by 91 Singaporean educators to date. Several international experts have been invited to conduct on-site faculty training in Singapore in the early phases. This ongoing journey continues to yield valuable insights and learning opportunities.

The most important lessons learned, with reference to NPT, are: (1) Stakeholders must be the central core of the process. Understanding their needs and concerns is essential, as well as their cooperation with strategies of implementation, backed by adequate resources. *Context* conditions of *Strategic intentions, Adaptive execution, Negotiating capacity and Reframing organizational logics* were all afforded and proved to be important; (2) Alignment was secured through a shared mental model, materialized in open-access resources (*Coherence*) that enhanced the process; (3) the credos "Think big - start small - move fast" and "Perfect is the enemy of good" helped to initiate early pilots to provide proofs of concept and reduce resistance to change by avoiding prolonged planning cycles (*Collective Action*) and (4) it proved important to first establish a service care model and review the regulatory framework for EPAs to be effective enablers to expand roles (*Reflexive Monitoring* and *relational integration as part of Collective Action*) .

3. **Taiwan.** The postgraduate CBME journey with Milestones and EPAs in Taiwan began in 2011 within the Taiwan Society of Emergency Medicine (TSEM), following a national

editorial questioning the value of the emergency medicine specialty. In response, TSEM launched a five-year implementation framework for CBME in 2012, developing a competency framework that combined milestones and EPAs to guide curriculum, content, and assessment design. Implementation strategies drew on Kotter's change management principles, on iterative pilot projects supported by national research grants, and on glocalized co-creation involving international collaboration and local consensus building to strengthen stakeholder engagement and professional ownership. In 2015, the Taiwan Society of Anesthesiologists (TSA) became the first follower, collaborating with TSEM's CBE champions to design a structured implementation plan. TSA mapped 24 ACGME-derived milestones to 8 EPAs and set four guiding priorities: Communication, Simplification, Technology, and Clinical relevance. Family Medicine and the Society of Respiratory Therapy soon followed, with Pediatrics and Otolaryngology joining thereafter. The movement rapidly expanded to other health professions, including Nutrition and Dietetics, Nursing, Pharmacy, Physical Therapy, Occupational Therapy, and Dentistry. This reform evolved through a bottom-up model, not mandated by national regulation but supported by government funding and policies under the Ministry of Health and Welfare. The process was symbolically described as the "Matsu Pilgrimage Model," reflecting Taiwan's cultural tradition of voluntary, collective participation. Much like pilgrims joining the Matsu procession in shared spirit and purpose, specialty societies and teaching hospitals advanced the CBME journey together, while governmental, research, and accreditation bodies provided structural support. This bottom-up yet top-supported dynamic reflected Taiwan's distinctive culture of democratic collaboration. In 2018, Dalin Tzu Chi Hospital, building on TSEM's framework, developed the EMYWAY CBME e-platform, integrating EPA-based workplace-based assessments, coaching feedback, and dashboards of entrustment levels. EMYWAY was adopted by the Joint Commission of Taiwan as a national technological support system in 2021, first piloted by the Taiwan Society of Otorhinolaryngology–Head and Neck Surgery and subsequently extended to other health professions.

Despite clear support for the implementation of CBME, various challenges were encountered along the way. At a national (macro) level, the creation of competency and EPA frameworks, with support of specialty associations and the Ministry of Health proved to be more laborious than anticipated. The strong emphasis on EPAs as an assessment tool disregarded the need for a cultural pedagogical shift. At the meso level, the introduction of programmatic assessment principles took quite some energy. Confusion arose about the division of labor between health care institutions and educational institutions and programs, either leading to doubling work, or tasks left unattended. The establishment of clinical competency committees (CCCs) was

fragmentary, and faculty development for program directors, supervisors and CCC members proved insufficient. At a micro level this led to lack of understanding about the EPA concept and to unclear expectations for assessment. The dual purpose of programmatic assessment data (for feedback and development versus for documentation and summative decision making) was not always well understood. A fundamental question arose: are trainees entitled to demand 'to be forgotten', that is, can or should initial substandard performance data of trainees be removed from their portfolios after a certain amount of time has passed?

Viewed from an NPT perspective, within the *Context* of Taiwan, the *transactional space* for improvement through CBME was available and reasonably clear, but the *adaptive execution*, and *reframing of organizational logistics* could have been better from the outset, with a clearer balance between top-down and bottom-up processes. The need to combine a change management strategy, focused on human behavior, with project management for technical aspects of curriculum development became clear during the process. The interdisciplinary, inter-institutional and international collaborations benefitted *Collective Action* and *interactional workability*. The dual purpose of assessment data is a rather universal wicked issue; it belongs to the conceptual understanding (*Coherence*) but also to s*kill-set workability* (*Collective Action*).

4. **North-America.** EPAs have been implemented as a component of CBME in Canada and the United States. However, their implementation processes have been quite different. The Royal College of Physicians and Surgeons of Canada (RCPSC) developed a unique model of CBME called "Competence by Design" (CbD), in which EPAs play a primary role for all specialties. There are several unique features of the Canadian context that influence CbD, and by extension, EPA implementation. First, the RCPSC oversees both program accreditation and individual certification (i.e. it serves a dual role), likely heightening trainees' sensitivity to individual assessment data. This may also create a tension between "ensuring competence" and "promoting excellence". Second, there is a strong culture of "community and consensus" that helps to empower trainees through collective representation and advocacy. Finally, in designing CbD, the RCPSC decided to make trainees responsible for collecting EPA assessments, in keeping with a spirit of trainee-centered education. While theoretically sound, these factors, when combined, have inadvertently created the impression of a high-stakes environment for direct observations, leading to unintended consequences such as incentives for residents to seek out specific supervisors or rotations for EPA assessment completion. In addition, other cultural factors have made individual institutions somewhat reluctant

to share detailed assessment data that could be used for national-level analytics. The implementation has faced some resistance, both from programs and from residents, and the RCPSC is currently considering system adaptations to address concerns.
In contrast, the U.S. system, with its separation of accreditation (ACGME) and voluntary Board certification, has resulted in a CBME framework with lower stakes individual assessments. EPA assessments in this context are not mandated at the national level, but are recommended for use as a supplementary tool. The development and implementation of EPAs, and the collection of individual EPA data, has been taken on by a few of the individual Boards who administer and confer individual physician certification. So far, the Boards of Pediatrics and Surgery have done so, and those of Family Medicine, Emergency Medicine, Internal Medicine, Orthopedics and Pathology are considering doing so. This lower-stakes approach, coupled with the existence of a national Milestones database, has allowed for national-level data analysis that can be used to determine whether CBME is meeting its original purpose.

From an NPT perspective, Reflexive Monitoring by the Royal College of Physicians and Surgeons of Canada, partly prompted by critical literature, has highlighted the need to strengthen conceptual understanding of EPAs (Coherence) and better tailor assessment procedures to individual specialties. This process is expected to enhance Cognitive Participation among stakeholders and improve workability and integration (Collective Action). In the United States, similar processes of Coherence-building and Cognitive Participation are occurring at the level of individual specialties or institutions, as illustrated by recent EPA initiatives in pediatrics and surgery.

5. **Switzerland.** In 2017, in a national effort, Switzerland was among the first countries to define the desired outcome of undergraduate medical education in terms of nine broad entrustable professional activities. In 2020, critical areas of implementation requiring support were identified: curricular design and governance, the assessment system and entrustment process, faculty development and change management, as well as procedures for curriculum mapping and the integration of an e-portfolio to support student learning. In 2021, the Swiss Institute for Medical Education (SIWF) charged with accreditation and certification in residency training and continuous professional development, initiated the introduction of CBME with EPAs at a national level. A dedicated EPA committee consisting of medical education experts was established to support specialties in their effort to develop EPA-based curricula. Transforming postgraduate medical education to CBME with EPAs began with pilots in cardiology and surgery in 2022. A previously established national teach-the-

teachers program included the topics of CBME and EPAs in their curriculum. While being of high-quality, the program’s scalability appeared limited and has not yet reached a broad audience (as of 2025). While progress has been made, the process is much different than anticipated and varies significantly across specialties. The impression is that stakeholders in the clinical environment often do not feel this innovation will benefit them, while it would cost time and effort. For many clinicians the value proposition is yet unclear, and reactions from the community are too often ‘we need a vision and clear path’, ‘we know better’, ‘we are confused’ or simply, ‘we disagree’. In addition, there was confusion in the community about the pathway towards implementation. While only pilots have started, some specialties interpreted this as full CBME implementation, a misunderstanding leading to tensions in the community.
In terms of NPT, at the macro level, there is a lack of *Coherence*, manifested as a superficial understanding of the functions and purposes of EPAs, often mistakenly viewed solely as an assessment tool. Communal specification, individual specification, and internalization remain underdeveloped. These are prerequisites for *Cognitive Participation*, which requires stronger initiation and legitimation among key clinical stakeholders. Although technological readiness currently supports workability (a *Collective Action* condition), broader communication and alignment across specialties are needed to strengthen interactional and contextual integration. *Reflexive Monitoring* is taking place at the macro level. For instance, the governing body SIWF now considers whether a too heavy focus on EPAs can realistically shift educational culture, or whether the pedagogical competence of key actors has been overestimated. At the meso level, teach-the-teacher programs have emphasized CBME and EPAs but have yet to be systematically evaluated, limiting evidence-based refinement. The *Contextual Preconditions*, particularly the prevailing cultural norms and political structures, remain significant challenges that must be addressed to advance normalization across the system.
